# 

**Published:** 1858-07

**Authors:** 


### RECEIPTS FROM JUNE 14th TO JULY 25th, 1858.

### Illinois.

| | | |
|---|---|---|
| Dr. R. C. Hamil, | vol. 1, C. J., 1858, | $2 |
| " J. F. Hamilton, | " " | 2 |
| " O. Q. Herrick, | " " | 2 |
| " Townsend Seely, | " " | 2 |
| " J. H. Hollister, | " " | 2 |
| " S. Wickersham, | " " | 2 |
| " Gore & Amerman, | " " | 2 |
| " V. L. Hulburt, | " " | 2 |
| " G. J. & Z. H. Whetmin, | " | 2 |
| " C. G. Smith, | " " | 2 |
| " J. Woodworth, | vol. 6 & 1 C. J. 1857-8, | 4 |
| " M. Parker, | vol. 1 C. J. 1858, | 2 |
| " P. Kirwan, | " " | 2 |
| " E. Richards, | " " | 2 |
| " C. H. Quinlan, | vol. 6 & 1 C. J. 1857-8, | 4 |
| " —— Lee, | vol. 1 C. J. 1858, | 2 |
| " J. H. Robinson, | " " | 2 |
| " F. R. Payne, | " " | 2 |
| " J. W. Green, | " " | 2 |
| " Josiah Slack, | " " | 2 |
| Dr. John Allen, | vol. 6, 1857, | $2 |
| " A. C. Buffum, | " | 2 |
| " C. C. Allen, | " | 2 |
| " C. D. Henton, | vol. 1 C. J. 1858, | 2 |
| " G. W. Rittell, | " " | 2 |
| " E. R. Willard, | vol. 6 & 1 C. J. 1857-8, | 4 |

### Indiana.

| | | |
|---|---|---|
| Dr. W. H. Sullivan, | vol. 1 C. J. 1858, | $2 |
| " C. Palmeter, | " " | 2 |

### Wisconsin.

| | | |
|---|---|---|
| Dr. J. S. Pashley, | vol. 1 C. J. 1858, | $2 |
| " E. G. Dyer, | " " | 2 |
| " Clark & Rice, | " " | 2 |
| " M. Waterhouse, | " " | 1 |
| " L. C. Bicknell, | " " | 2 |

### Iowa.

| | | |
|---|---|---|
| Dr. S. M. Dunn, | vol. 3, 1854, | $2 |

